# Supplementary material for: Implications of Crop Rotation and Fungicide on Fusarium and Mycotoxin Spectra in Manitoba Barley, 2017–2019
Source: Toxins (Basel). 2022 Jul 6;14(7):463. doi: 10.3390/toxins14070463 (PMC9319603; doi:10.3390/toxins14070463)
Supplement: Supplementary file 1 [file toxins-14-00463-s001.zip › toxins-1787912-supplementary.pdf]

Supplementary Materials: Implications of Crop Rotation and Fungicide on *Fusarium* and Mycotoxin Spectra in Manitoba Barley, 2017-2019

Table S1. *Fusarium* Species/Chemotype-specific Primers with Sequences.

Target *Fusarium* spp.	Primer Name	Sequences (5′-3′)	References
**A.** Conventional PCR			
F. poae	Fp82F	CAAGCAAACAGGCTCTTCACC	[1]
	Fp82R	TGTTCCACCTCAGTGACAGGTT	
F. graminearum	Fg16F	CTCCGGATATGTTGCGTCAA	
	Fg16R	GGTAGGTATCCGACATGGCAA	
F. sporotrichioides	FspF	CGCACGTATAGATGGACAAG	
	FspR	GTCAGAAGAGACGCATCCGCC	
F. avenaceum	FaF	CAAGCATTGTCGCCACTCTC	
	FaR	GTTTGGCTCTACCGGGACTG	
F. equiseti	FEF1	CATACCTATACGTTGCCTCG	
	FER1	TTACCAGTAACGAGGTGTATG	
**B.** Quantitative PCR			
F. poae	FpA51F	ACCGAATCTCAACTCCGCTTT	[2]
	FpA98R	GTCTGTCAAGCATGTTAGCACAAGT	
F. graminearum	FgB397F	CCATTCCCTGGGCGCT	
	FgB411R	CCTATTGACAGGTGGTTAGTGACTGG	
F. sporotrichioides	FspA18F	GCAAGTCGACCACTGTGAGTACA	
	FspA85R	CTGTCAAAGCATGTCAGTAAAAATGAT	
**C.** Chemotype Detection			
F. graminearum	3CON	TGGCAAAGACTGGTTCAC	[3]
	3NA	GTGCACAGAATATACGAGC	
	3D15A	ACTGACCCAAGCTGCCATC	
	3D3A	CGCATTGGCTAACACATG	
F. poae	nivPF	TATCCTTGCATGGCAATGCC	[4]
	nivPR	AAATGGCGATACGAGTATTGA	

Table S2: Precursor and Product Ions (m/z), Retention Times, and Recoveries of Various Mycotoxins from Barley Grains

Mycotoxin (Abbreviation)	Precursor Ion (m/z)	Product Ion (m/z)	Retention Time (min)	Recoveries Mean ± SD
Deoxynivalenol (DON)	297.1333	203.1065; 231.1014	4.39	91 ± 7
Diacetoxyscirpenol (DAS)	384.2017	307.1554; 205.0857	8.67	95 ± 6
Nivalenol (NIV)	313.1282	175.0752; 229.0858	3.36	81 ± 6
Beauvericin (BEA)	801.4433	244.1327; 262.1432	13.82	47 ± 9
HT-2 Toxin (HT-2)	442.2435	263.1277; 215.1067	9.76	94 ± 5
T-2 Toxin (T-2)	484.2541	215.1067; 185.0960	10.54	119 ± 6
Enniatin A (ENN A)	699.4903	682.4638; 210.1488	14.28	61 ± 6
Enniatin A1 (ENN A1)	685.4746	668.4480; 210.1488	14.11	63 ± 7
Enniatin B (ENN B)	657.4433	640.4166; 196.1331	13.68	55 ± 4
Enniatin B1 (ENN B1)	671.4903	654.4324; 196.1331	13.91	57 ± 3

[a] Number of spiked samples, $n = 9$

Table S3 Climatic Properties (Temperature and Precipitation) across Manitoba Crop Districts in July during the FHB Survey (2016-2019).

Manitoba crop districts	July temperature (°C)				July precipitation (mm)			
	2016	2017	2018	2019	2016	2017	2018	2019
Central (CMB)	19.4	19.8	20.3	19.8	106.6	37.6	50.2	63.4
Southwest (SWMB)	18.8	19.4	19.4	19.6	85.4	40.5	34.2	55.6
Eastern (EMB)	20.3	20.9	21.1	20.1	88.2	56.7	44.3	61.3
Northwest (NWMB)	18.6	19.9	19.7	19.2	84.5	31.2	42.1	40.6
Interlake (INMB)	18.8	18.8	19.4	19.1	52.2	72.3	71.2	74.3
Provincial mean	19.2	19.8	20.0	19.6	83.4	47.7	48.4	59.0

Note: Manitoba local climate data retrieved from www.gov.mb.ca/agriculture/weather/ and https://climate.weather.gc.ca/historical_data/search_historic_data_e.html

Figure S1. Geographical Locations of Barley Fields in Manitoba Crop Districts Surveyed from 2017 to 2019.

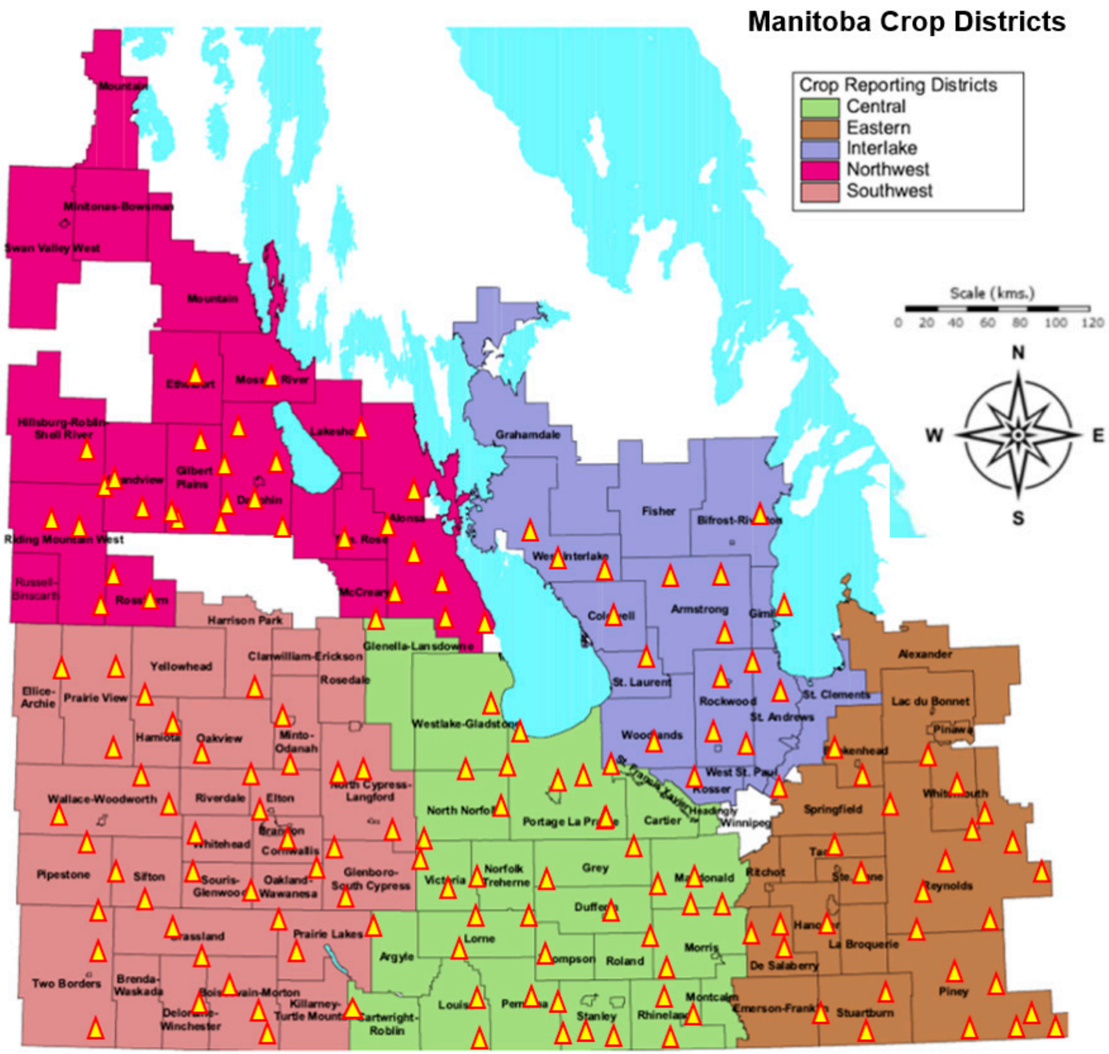

Reference:

1. Demeke, T.; Clear, R.M.; Patrick, S.K.; Gaba, D. Species-specific PCR-based assays for the detection of Fusarium species and a comparison with the whole seed agar plate method and trichothecene analysis. *International Journal of Food Microbiology* **2005**, *103*, 271-284.
2. Nicolaisen, M.; Supronienė, S.; Nielsen, L.K.; Lazzaro, I.; Spliid, N.H.; Justesen, A.F. Real-time PCR for quantification of eleven individual Fusarium species in cereals. *Journal of Microbiological Methods* **2009**, *76*, 234-240.
3. Ward, T.J.; Clear, R.M.; Rooney, A.P.; O'Donnell, K.; Gaba, D.; Patrick, S.; Starkey, D.E.; Gilbert, J.; Geiser, D.M.; Nowicki, T.W. An adaptive evolutionary shift in Fusarium head blight pathogen populations is driving the rapid spread of more toxigenic *Fusarium graminearum* in North America. *Fungal Genetics and Biology* **2008**.
4. Dinolfo, M.I.; Barros, G.G.; Stenglein, S.A. Development of a PCR assay to detect the potential production of nivalenol in Fusarium poae. **2012**, *332*, 99-104.
